# Extradural spinal melanoma: is it primary or metastatic? A case report with a brief review of literature

**DOI:** 10.1186/s13000-024-01475-4

**Published:** 2024-03-20

**Authors:** Raghav Kapoor, Anurag Mehta, Anila Sharma, Shrinidhi Nathany, Himanshi Diwan, Divya Bansal

**Affiliations:** Rajiv Gandhi Cancer Institute and Research Centre, New Delhi, India

## Abstract

Melanocytic lesions involving the central nervous system are extremely rare and pose a diagnostic challenge owing melanoma being the third most common malignancy metastasizing to the spine. Morphology and immunohistochemistry are identical in both primary and secondary cases, and hence may not help in rendering a final diagnosis. Molecular alterations involving melanomas of the spine and melanomas elsewhere are distinct and help establish the appropriate diagnosis. We report an interesting case where molecular profiling of the tumor tissue helped render the final diagnosis.

## Introduction

Pigmented intramedullary/extradural tumors of the central nervous system (CNS) are exceedingly rare, with only a few anecdotal reports in literature [1]. A pigmented intramedullary/extradural lesion on histopathologic evaluation warrants consideration of several differential diagnoses, including primary melanoma, melanoma metastases, melanocytic ependymoma, melanocytoma and melanotic schwannoma [2]. Melanoma is the third most common malignancy resulting in brain metastases, hence differentiating primary from secondary is essential to render optimal treatment [3]. The same cannot be obtained by employing an immunohistochemistry (IHC) panel (S100P, HMB-45 and Melan-A) as one or all of these will be positive in both primary and secondary malignancies [3].

Distinguishing primary melanoma of the CNS and metastatic melanoma by pathological examination is almost impossible as both of these are histologically identical, and display cellular atypia, numerous mitoses, and often demonstrate unequivocal tissue invasion or coagulative necrosis [3]. Melanomas of the skin are known to harbor *BRAF* p.V600E mutations, which can be detected on both IHC and molecular testing. A few cases also are known to harbor mutations in *NRAS* and *HRAS* genes [3]. However, melanomas of CNS do not show this canonical genetic alteration, adding to the diagnostic dilemma.

Activating mutations in *GNAQ g*ene (G alpha q gene) especially residing at codon 209 have been reported in uveal melanomas. Recently reports have emerged of similar codon 209 mutations in primary intramedullary melanomas, involving the spine [4]. Identification of codon 209 mutation of *GNAQ* can thus be used to establish diagnosis of primary central nervous system melanoma in the appropriate context. While it may be possible to interrogate codon 209 by single gene assays, a broader next generation panel can be more informative and help exclude other differential diagnoses. Other mutations in *GNAQ* have also been reported in circumscribed choroidal hemangiomas [5].

We report an interesting case, where the diagnostic dilemma in a melanocytic lesion of the spine was solved using NGS based testing.

## Case report

**Fig. 1:**
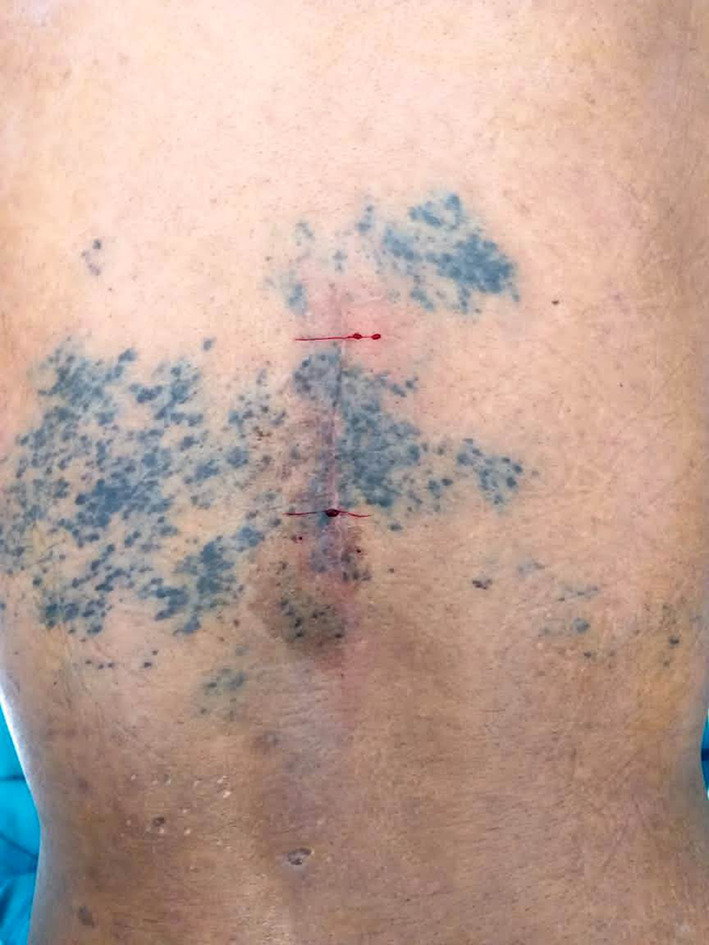
Pigmented lesion on the skin surface of the dorsal spine

**Fig. 2:**
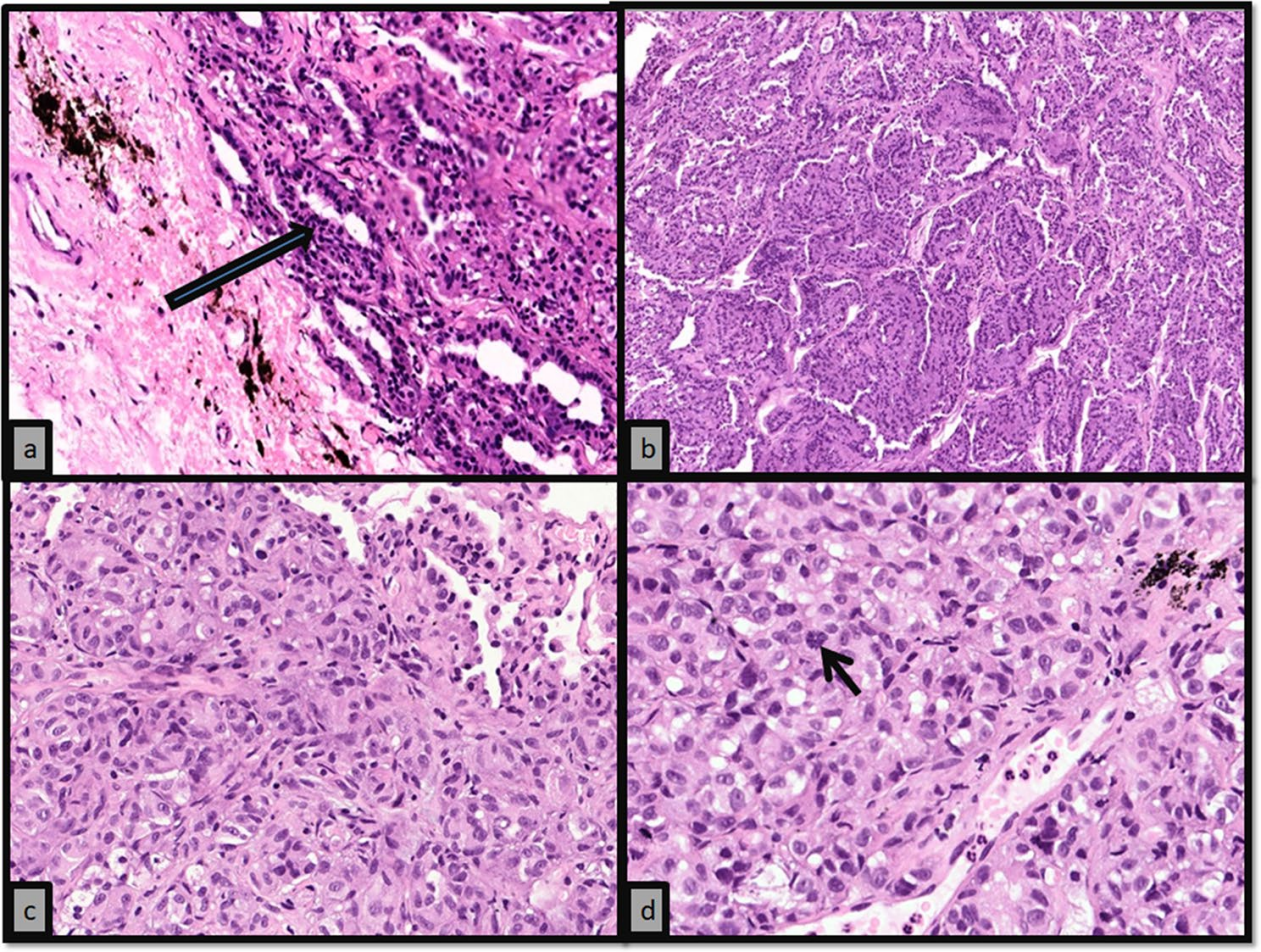
(**a**) Extradural lesion showed a tumor arranged in predominantly glandular pattern (blue arrow) with surrounding fibrocollagenous tissue showing melanin deposition (H&E; X100); (**b**) Extradural tumor showed varied morphological patterns like glandular, papillaroid and nested (H&E; X40); (**c**) Lung parenchyma infiltrated by a tumor arranged in nested pattern (H&E; X200); (**d**) The tumor cells had eosinophilic cytoplasm with a prominent nucleolus and significant mitosis (arrow) (H&E; X400)

**Fig. 3:**
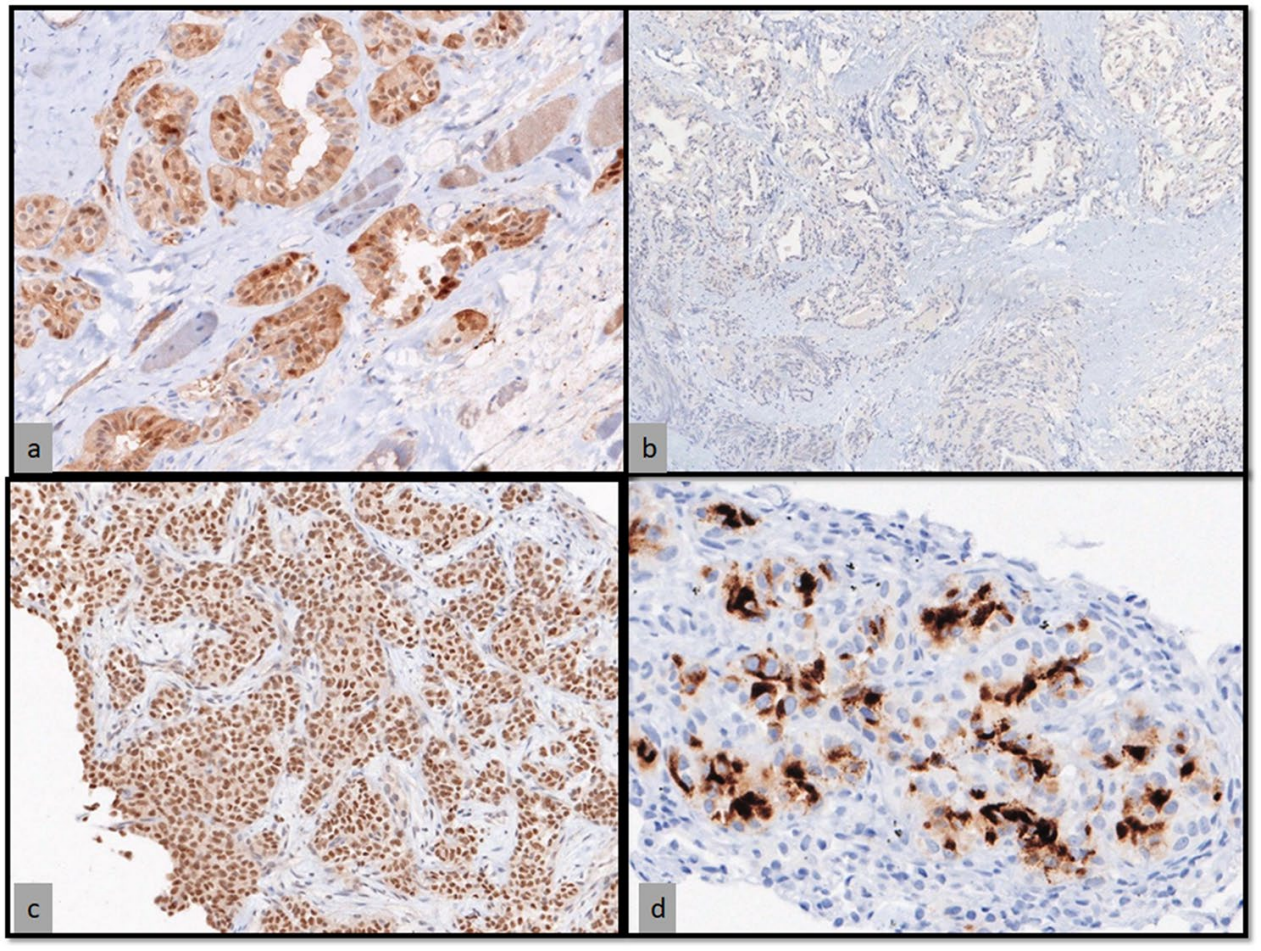
(**a**) S-100 shows both cytoplasmic and nuclear immunoexpression in tumor cells (DAB; X100); (**b**) CK was negative in the tumor cells (DAB; X40); (**c**) SOX10 shows nuclear expression in tumor cells (DAB; X100); (**d**) HMB-45 shows cytoplasmic expression in tumor cells (DAB; X100)

**Fig. 4:**
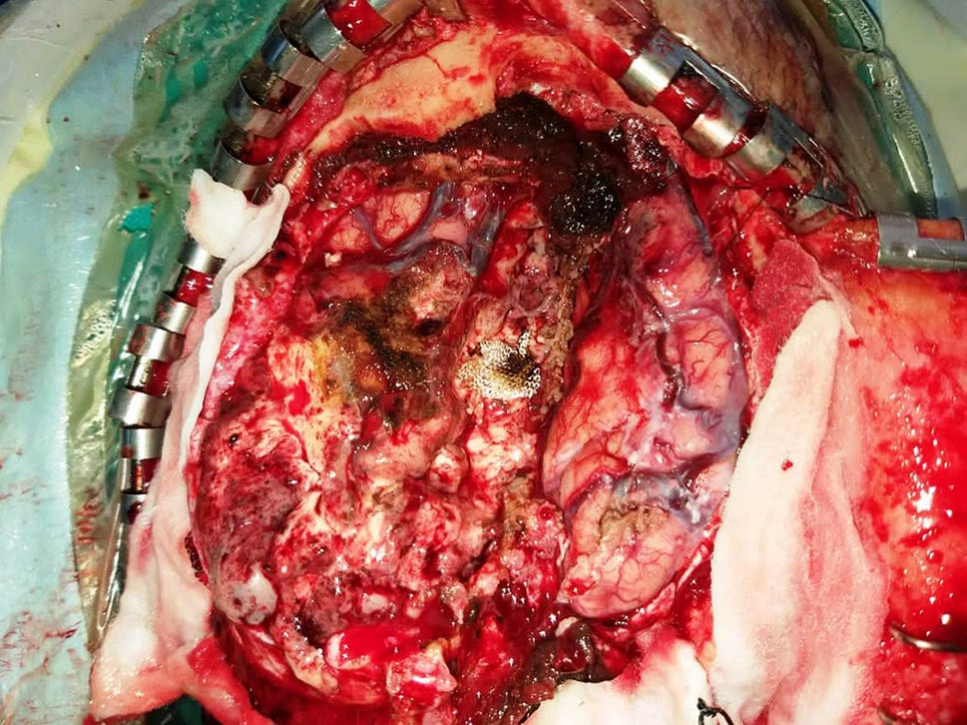
Intraoperative image showing the pigmented dural surface

A 51 year old male patient presented to our outpatient facility with complaints of lower backache associated with stiffness and difficulty in walking of 2 months duration. He was operated elsewhere for an adherent extradural tumor involving the D8-D10 dorsal vertebrae. Pathologic examination done elsewhere was suggestive of a low grade neuroepithelial tumor. There was no medically relevant past or family history. On examination, the patient had a pigmented lesion on the dorsal spine (Fig. 1), present since birth with no changes in size/texture /contour and was later diagnosed as neurocutaneous melanosis. MRI of the dorsal spine revelaed a residual/recurrent epithelial soft tissue mass extending bilaterally to D7-D10 with cord compression. FDG PET-CT showed an extradural lesion in dorsal spine, with a soft tissue nodular lesion in right lower lobe of lung with multiple enlarged mediastinal lymph nodes. CT guided biopsy of the lung lesion was done which on histopathology revealed a tumor arranged in variably sized nests with tumor cells showing pale eosinophilic to vacuolated cytoplasm, with irregular convoluted nuclei and inconspicuous to conspicuous nucleoli. Mitotic activity was significant (Fig. 2a-d). On immunohistochemistry, the tumor cells were positive for S100P and SOX-10 and focally positive for HMB-45 and Melan A. Tumor cells were negative for CK, EMA, SMA, TTF1, PAX8, GATA3 and SALL4. (Fig. 3a-d) IHC for BRAF V600E was also negative. A diagnosis of a melanocytic lesion was rendered with possibilities of metastatic melanoma, primary melanoma of spine, and a clear cell sarcoma. In view of recurrent mass lesion, a re-exploration surgery was performed for D8-D9 and laminectomy of D7-D10 was done. A gross total removal of extradural lesion was done. Intraoperatively, the dura was also found to be pigmented (Fig. 4). The patient tolerated the surgery well.

A next generation sequencing assay was performed on the tumor tissue excised from the D8-D10 region, using the Oncomine Focus Assay, comprising of 52 genes. The prepared libraries were quality checked and run on Ion Torrent S5 sequencer. The NGS assay revealed a missense mutation p. Q209P in exon 5 of the *GNAQ* gene bearing the coordinates chr9:80409487 with a variant allele frequency of 37.83%. The tumor fraction on the section was 70%. This variant was predicted pathogenic using ACMG/AMP guidelines [6] and also as a gain-of-function mutation in the OncoKB databases [7]. This variant has been reported in COSMIC [8] and ClinVar [9] databases in tumor samples from uveal and primary intramedullary melanomas.

Post surgery the patient was mobilized by optimal physiotherapy and is currently in fair general condition and is due for first follow up.

## Discussion

This is an interesting case where molecular diagnostics as an ancillary technique helped render a final diagnosis. Primary spinal melanomas are rare neoplasms, derived from scattered melanocytes of the leptomeninges [1]. These melanocytes are derivatives of the neural crest cells and are frequently found in the recesses of the sulci at the base of the brain, around the brain stem and the upper part of the spinal cord. Evidence from literature suggests that these are a heterogeneous group of tumors in terms of molecular alterations, which are distinct from melanomas occurring at other sites. The incidence is exceedingly rare and has been reported to occur in 0.005 cases per 100,000 population [10].

Clinically these are reported in fifth decade as seen in our case, with symptoms of motor weakness and loss of bowel/bladder controls. The diagnostic modality for visualization to be considered is an MRI along with histopathologic evaluation. Immunohistochemically, melanocytic markers may or may not be strongly expressed, and also is not sufficient to differentiate primary versus a secondary melanoma.

The differential diagnosis to be considered include metastatic melanomas from elsewhere, clear cell sarcoma, melanocytomas, melanocytic ependymoma and melanotic schwannomas. A detailed review of these is depicted in Table 1 [2, 3, 11].

**Table 1:** Differential diagnoses for primary spinal melanoma with differentiating features

Features	Primary spinal melanoma	Metastatic Melanoma	Melanocytoma	Melanotic Schwannoma	Clear cell Sarcoma
**Age/Sex**	50–70/males	Any age	5th decade/males	20–50/males	30–70/males
**Site**	Spine/extradural intramedullary	Any site	Cervical, thoracic spine, posterior fossa, Meckels cave	CNS: thoracic spine Perivertebral tissue	
**Imaging**	Extradural lesion	Nothing specific		Extradural	
**Gross**	Soft	Pigmented		Soft, pigmented	Firm, grey, gritty
**Microscopy** • Cellular atypia • Tissue invasion • Necrosis • Mitoses • Pigment	Present Present Coagulative Numerous +	Present Present + Numerous +/-	Variable/- Rare Rare Rare +	Variable Rare + /- +/- +	Present Present Present Present + in 60% cases
**Immunohistochemistry** • CK • TTF1 • EMA • HMB45 • S100P • Melan-A • Ki-67 • Others	- - - + + + 8–10% MITF, MART-1	- - - + + + 8–10% ----	- - - + + - 1–4% MITF, MART-1 NKIC3	- - - + + + -- GFAP±	+ - - + + + 3–4% MITF, MCAM
**Molecular Alterations**	*GNAQ* (37% cases)	*BRAF V600E/NRAS/HRAS*	*PRKAR1A*	*PRKAR1A*	t(12;22)
**Treatment preferred**	Surgical Excision	Excision + chemotherapy	Excision	Excision	Excision + radiation
**Prognosis**	Benign to indolent, rarely aggressive	Depends on stage	Indolent	Indolent to aggressive	Indolent to Aggressive

Oncogenic mutations in *BRAF, NRAS HRAS* genes have been described commonly in melanomas of skin and other sites, however, the same at this site has been conspicuously absent. Alternative genetic mechanism activating the MAPK pathway involves mutations in the *GNAQ* gene which have been described in uveal melanoma, blue nevi, Nevi of Ota and Spitz nevi. Codon 209 mutation as encountered in our case has also been described in 37% cases of primary CNS melanomas [4], including the spine, in a series of 19 such cases reported by Vandevelde et al. The most commonly reported mutation in *GNAQ* is p.Q209L in primary spinal melanomas. Our case had a p.Q209P which also has been predicted to have the same effect as the reported variant. Identification of *GNAQ* mutations have therapeutic implications as there are clinical trials evaluating the use of MEK inhibitors [2].

This report highlights the importance of molecular testing as an essential tool in the diagnoses of common tumors in rare locations. Sometimes, canonical mutations clinch the diagnosis as seen in this case, which helps the clinician to render optimal treatment to the patient.

## Data Availability

Not applicable.
